# Comparison of high‐dose‐rate intracavitary brachytherapy dosimetry with and without anesthesia in patients with cervical carcinoma

**DOI:** 10.1120/jacmp.v15i2.4670

**Published:** 2014-03-06

**Authors:** Daya N. Sharma, Pritee Chaudhari, Seema Sharma, Leena Gupta, Pandjatcharam Jagadesan, Goura K. Rath, Pramod K. Julka

**Affiliations:** ^1^ Department of Radiation Oncology All India Institute of Medical Sciences New Delhi India

**Keywords:** cervix, intracavitary brachytherapy, dosimetry, anesthesia

## Abstract

This study compares the dosimetry of high‐dose‐rate intracavitary brachytherapy (HDR‐ICBT) performed with and without general anesthesia/spinal anesthesia (GA/SA) in patients with cervical carcinoma. We retrospectively retrieved the records of 138 HDR‐ICBT applicator insertions performed in 46 patients: 69 performed with GA/SA (anesthesia group known as AG) in 23 patients, and 69 performed without GA/SA (nonanesthesia group known as NAG) in 23 patients. The intracavitary brachytherapy (ICBT) application was done with central tandem and two vaginal ovoids. For each ICBT plan, a high‐dose‐rate (HDR) dose of 7 Gy was prescribed to point A. From each plan, the doses to Point B right $\left(B_{R}\right)$, Point B left $\left(B_{L}\right)$, bladder and rectal reference points (${\text{Bladder}}_{\text{ref}}$ and ${\text{Rectal}}_{\text{ref}}$) were recorded and compared in the two groups. Student's t‐test was applied to find out the significance of difference. The two groups were comparable in terms of demography and clinical characteristics. Mean Point $B_{L}$ doses in AG and NAG were 1.89 Gy (27% of Point A dose) and 1.82 Gy (26% of Point A dose), respectively. Mean Point $B_{R}$ doses in AG and NAG were 1.91 Gy (27% of Point A dose) and 1.85 Gy (26% of point A), respectively (p‐value 0.7). The mean dose to ${\text{Bladder}}_{\text{ref}}$ in AG and NAG was 5.03 Gy and 4.90 Gy, respectively (p‐value 0.6). The mean dose to ${\text{Rectal}}_{\text{ref}}$ was significantly higher in AG than NAG (5.09 Gy vs. 4.49 Gy, p‐value 0.01). Although based on conventional 2D dosimetry planning, our study has demonstrated that avoiding GA/SA does not result in inferior HDR‐ICBT dosimetry.

PACS number: 87.53.Jw, 87.50.cm

## I. INTRODUCTION

Intracavitary brachytherapy (ICBT) is an important treatment in the management of cervical carcinoma.^(1)^ The standard ICBT application consists of insertion of a central tandem in the uterine cavity and two ovoids in the vagina, and delivering the brachytherapy treatment by feeding sources through applicator. It is a mean to deliver the required dose of brachytherapy to cervical and parametrial region with relative sparing of the adjoining normal structures.^(2)^ Though often combined with external‐beam radiation therapy (EBRT) in a curative setting, it can be used alone in very early stage^(3)^ and rarely as palliative or haemostatic treatment^(4)^ to relieve the severe bleeding from cervical tumor not controlled by EBRT. It can be practiced in the form of low‐dose‐rate (LDR) or pulsed‐dose‐rate (PDR) or more often HDR. HDR treatment is completed within minutes and applicator is removed within 2‐3 hours after the applicator insertion; but requires 2‐6 fractions.^(5)^

Various forms of anesthesia have been used for inserting the HDR‐ICBT applicator: general anesthesia/spinal anesthesia (GA/SA), paracervical block and conscious sedation (CS).^(6)^ Different institutions choose the method of anesthesia which suits its patients and the institute. Though GA/SA provides good analgesia and muscle relaxation, it has shown to be associated with higher complication rate.^(7)^ CS, on the other hand, is simple and convenient to practice, but may cause pain, discomfort, and poor muscle relaxation. This might lead to improper ICBT applicator placement and poor vaginal packing, resulting in higher doses to organs at risk (OAR), thus compromising dosimetry; however, no such study exists in the literature to date. We conducted a study to compare the HDR‐ICBT dosimetry with and without GA/SA in patients with cervical carcinoma to find out if the lack of GA/SA affected the dosimetry.

## II. MATERIALS AND METHODS

Till the end of year 2007, our institutional practice was to administer GA/SA for performing HDR‐ICBT for cervical cancer patients. Since 2008, we have stopped giving GA/SA and almost all HDR‐ICBT applicator insertions are performed under CS. The present study is based on the analysis of dosimetric data of 138 HDR‐ICBT plans (46 patients), 69 with GA/SA and 69 without GA/SA, to find out if the lack of GA/SA yielded inferior dosimetry. We retrieved the ICBT dosimetry data of 23 patients (at random) who underwent three HDR‐ICBT applications (once a week application, each application delivering a single fraction of 7 Gy to point A) under GA/SA in the year 2007. Similarly, data of 23 patients were retrieved in year 2008 who underwent similar treatment without GA/SA.

The treatment consisted of whole‐pelvis EBRT with a dose of 50.4 Gy in 28 fractions over 5.5 weeks by the four‐field box technique with concurrent weekly cisplatin. After the course of EBRT, patients were assessed and found eligible for standard HDR‐ICBT application.

### A. ICBT applicator insertion

Twenty‐three patients underwent 69 HDR‐ICBT applications under GA/SA and the other 23 underwent equal number of applications without GA/SA. Pre‐anesthetic checkup was done for all anesthesia group (AG) patients and the anesthetic team decided the type of anesthesia, GA or SA, to be administered. The patients were admitted a day prior in the radiotherapy indoor ward and premedication was given as instructed by the anesthetist. The applicator insertion was done in the operating room in the lithotomy position. After cleaning and draping, Foley's catheter was inserted into the urinary bladder and the bulb was inflated with 7 cc saline. After a thorough pelvic examination, uterine sounding was done and length of uterine cavity was measured. No Smitt sleeve was placed in the cervical opening. ICBT insertion was performed using Nucletron CT/MRI compatible applicator consisting of central tandem and two ovoids (Nucletron, Elekta AB, Stockholm, Sweden). Size of the ovoids was chosen according to the vaginal space. No rectal retractor was used. Adequate vaginal packing with roll gauze was done to increase the distance between applicator and bladder and rectum. The applicator was immobilized with T‐bandage. A rectal marker was also placed to better visualize the rectum. The ICBT applicator insertion was similar in nonanesthesia group (NAG) patients, except the CS which was administered instead of GA/SA. For the CS, pentazocine 15‐30 mg and phenergan 25 mg was given intravenously 20 min before the applicator insertion.

### B. ICBT planning

**Figure 1:**
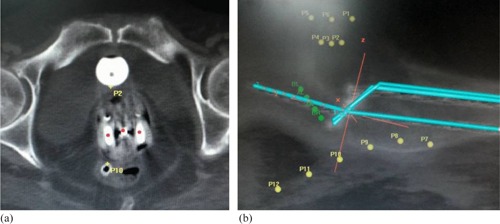
CT Scan images showing the marking of bladder and rectal points: (a) axial CT image showing ICBT applicator with central tandem and two ovoids where the bladder point can be seen on the posterior surface of Foley's bulb and the rectal point can be seen on the anterior surface of marker tube inserted into the rectum; (b) sagittal CT image showing the reconstructed ICBT applicator, multiple bladder points anteriorly and rectal points posteriorly.

**Figure 2:**
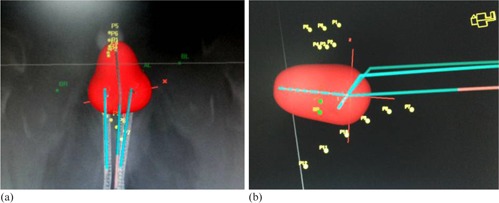
CT scan showing pear‐shaped isodose distribution on axial (a) and sagittal (b) images.

After every applicator insertion, the patients were shifted to CT scan room for imaging. A planning CT scan of the whole pelvis was done with slice thickness of 3 mm. The images were then sent through DICOM‐RT to brachytherapy planning system (PLATO planning system, version 14.1; Nucletron, The Netherlands). The applicator was reconstructed and the points A and B were marked, per International Commission on Radiation Units and Measurements (ICRU) Report 38.^(8)^ Bladder and rectal points were marked on axial CT images, as shown in Fig. 1. Multiple points were marked along the posterior surface of the Foley's bulb visible on serial CT images and the posterior most point or the point receiving the highest dose was labeled as bladder reference point (${\text{Bladder}}_{\text{ref}}$ point). Similarly, multiple points at a distance of 9 mm were placed along the anterior most surface of the rectal marker tube starting from anorectal junction up to recto‐sigmoid junction. The closest point to central tandem or the point receiving the highest dose was labeled as rectal reference point (${\text{Rectal}}_{\text{ref}}$ point). Multiple active dwell positions were chosen in central tandem and ovoids at an interval of 2.5‐5.0 mm to create a pear‐shaped dose distribution (Fig. 2). A prescription dose of 7 Gy was normalized to point A and the relative dose to the ${\text{Bladder}}_{\text{ref}}$ and ${\text{Rectal}}_{\text{ref}}$ points and point B were then calculated. If required, manual optimization was done to keep the ${\text{Bladder}}_{\text{ref}}$ and ${\text{Rectal}}_{\text{ref}}$ point doses below 80% of point A dose. For every individual insertion, a fresh optimum plan was generated and doses to various above mentioned points were recorded.

### C. HDR‐ICBT treatment

After the plan approval, patients were taken to brachytherapy suite ((microSelectron; Nucletron, Elekta AB, Stockholm, Sweden) HDR remote after loading unit using Ir‐192 radioactive source with initial activity of 10 Ci) for treatment delivery. Treatment was delivered with a prescription dose of 7 Gy to point A (one fraction each insertion). The applicator was removed immediately after the completion of treatment. A total of three HDR‐ICBT applications were performed for each patient at weekly interval.

### D. Statistical analysis

From each ICBT plan, the doses to Point B right $\left(B_{R}\right)$, Point B left $\left(B_{L}\right)$, ${\text{Bladder}}_{\text{ref}}$ point, and ${\text{Rectal}}_{\text{ref}}$ point were recorded and compared in the two groups (AG and NAG). Student's *t*‐test was applied to find out the significance of the difference. P‐value of <.05 was considered significant.

## III. RESULTS

Table 1 shows the various demographic and clinical characteristics of the patients. The two groups were well matched with respect to these attributes. There was one plan for each insertion. Thus a total of 138 plans were generated, 69 in each group. Dosimetric comparison of the two groups is presented in Table 2. Mean Point $B_{R}$ doses in the AG and NAG were 1.91 Gy (27% of Point A dose) and 1.85 Gy (26% of point A), respectively, with a p‐value of 0.7. Mean Point $B_{L}$ doses in the AG and NAG were 1.89 Gy (27% of Point A dose) and 1.82 Gy (26% of Point A dose), respectively. The mean dose to ${\text{rectal}}_{\text{ref}}$ point was significantly higher in the AG than NAG (5.09 Gy vs. 4.49 Gy, p‐value 0.01). The mean dose to ${\text{bladder}}_{\text{ref}}$ point in the AG and NAG was 5.03 Gy and 4.90 Gy, respectively, but the difference was not statistically significant (p‐value 0.6).

**Table 1:** Patient characteristics

*Attribute*	*Anesthesia Group (AG)*	*Nonanesthesia (NAG)*
Median Age (Year)	49	48
FIGO Stage (No. of patients)		
II	11	10
III	12	13
Size of primary tumor (cm)		
Average	4.2	4.0
Range	2.5−6.0	2.0−6.0
Median EBRT dose (Gy)	50.4	50.4
Median duration of treatment (days)	61	57
ICBT		
Average applicator insertion time in OT (min)	40	25
Dose per fraction (Gy)	7	7
Length of uterine cavity (cm)	5	5
Median ovoid size	Medium	Medium

FIGO=International Federation of Gynecology and Obstetrics; cm=centimeter; Gy=Gray; EBRT=external beam radiation therapy; OT=operation theater; ICBT=intracavitary brachytherapy.

**Table 2:** Dosimetric comparison with and without general/spinal anesthesia. Figures in parentheses represent standard deviation

*Variable*	*Anesthesia Group (AG)*	*Nonanesthesia Group (NAG)*	*p‐value*
No. of ICBT plans	69	69	–
Dose to Point A (Gy)	7	7	–
Mean Dose to Point $B_{R}$ (Gy)	1.91 (0.22)	1.85 (0.13)	0.07
Mean Dose to Point $B_{L}$ (Gy)	1.89 (0.18)	1.82 (0.14)	0.01
Mean Dose to ${\text{Rectal}}_{\text{ref}}$ point (Gy)	5.09 (1.64)	4.49 (0.83)	0.01
Mean Dose to ${\text{Bladder}}_{\text{ref}}$ point (Gy)	5.03 (1.89)	4.90 (1.38)	0.6

ICBT=intracavitary brachytherapy; Gy=Gray.

## IV. DISCUSSION

The use of ICBT for gynecological malignancy was first reported by Margaret Cleaves in 1903.^(9)^ Since then, ICBT has been the anchor of brachytherapy and represents an important milestone in the history of radiotherapy. It is a frequently practiced procedure in countries where cervical carcinoma is very common. Though ICBT has been vastly studied and constitutes an integral portion of cervical cancer treatment, there are limited studies^7^, ^10^, ^11^, ^12^, ^13^, ^14^ regarding its anesthetic perspective. More so, the ICBT anesthesia studies have dealt with the efficacy and the toxicity aspect, but rarely with dosimetric perspective. To the best of our knowledge, there is only one study^(15)^ in the literature so far, correlating the anesthesia and the ICBT dosimetry, and ours is the second such study.

American Brachytherapy Society (ABS) guidelines^16^, ^17^ suggest that different anesthetic forms may be used depending on the comfort of the patient. A Gynaecological Cancer Intergroup (GCIG) survey^(6)^ revealed that for ICBT applicator insertion, 46% patients received GA, 27% SA, 28% CS, and 14% oral pain medication.

It is generally assumed that lack of GA/SA may result in inferior dosimetry due to improper ICBT applicator placement and poor vaginal packing.^(17)^ The results of our present study have shown that performing HDR‐ICBT without GA/SA does not provide inferior dosimetry. The mean bladder dose in the two groups was found comparable in our study. On the contrary, mean rectum dose in AG patients was significantly higher than NAG patients. This was an unexpected finding in our study and we do not attribute any specific reason for this. Anker et al.^(15)^ in their study also unexpectedly observed significantly higher OAR (bladder) dose when intravenous anesthesia was used and cited no particular reason. They evaluated the dosimetry of 179 HDR‐ICBT procedures performed in 31 patients to investigate the effects of patient characteristics and treatment variables on HDR‐ICBT dosimetry. Fourteen patients were given intravenous anesthesia and the other 17 were managed by oral analgesics. Doses to various points of interest (per ICRU Report 38), including OARs and the pelvic sidewall, were assessed. Like our study, they also found that intravenous anesthesia usage was not correlated with improved dosimetry.

Recently, Bhanabhai et al.^(18)^ studied the effectiveness of CS for pain control during HDR‐ICBT using a ring‐and‐tandem applicator system for patients with cervical cancer. A total of 57 procedures were performed in 20 patients. They demonstrated good pain control with CS, and concluded that HDR‐ICBT procedure can be successfully performed under CS but, unlike us, they did not evaluated dosimetry.

A study by Lim et al.^(7)^ reported the complications arising from different forms of anesthesia used for HDR‐ICBT. A total of 84 fractions of HDR brachytherapy were delivered to 18 eligible patients. Eight patients were treated with 3 fractions of 6 Gy each to Point A and 10 patients were given 6 fractions of 5.3 Gy to Point A. Nineteen fractions were given under GA, 41 under topical anesthesia and sedation, five under paracervical nerve block, and 19 under CS only. GA had significantly more complications than topical anesthesia or CS (p<0.001). To avoid the risk of anesthesia related complications, Chen et al.^(10)^ studied the use of 10% lidocaine vaginal application for HDR‐ICBT in 40 patients and found it safe. They concluded that 10% lidocaine solution can significantly reduce the degree of painful sensation during HDR‐ICBT.

The overall duration of HDR‐ICBT application procedure under GA/SA is relatively longer due to extra time required for administering GA/SA (Table 1). In a high volume center, administering GA/SA for HDR‐ICBT cases may consume a significant proportion of operation theater time. This might overburden the resources, resulting in prolonging the waiting list and limiting the number of cases. Prolonged overall treatment time has been proved to adversely affect the outcome of cervical cancer patients.^(19)^ Since ours is a high volume center performing about 20‐25 HDR‐ICBT applicator insertions in a week, administering GA/SA would limit the number of ICBT applications in a given day. This was our main reason for switching over from GA/SA to CS, though we were skeptical about the inferior dosimetric outcome. The results of our analysis have removed this skepticism. In our current practice, we rarely use GA/SA, and only for patients who are not cooperative enough and who feel pain/discomfort due to HDR‐ICBT applicator insertion.

We realize the limitations of our study: 1) retrospective and nonrandomized nature; 2) no attempt to evaluate the pain and discomfort due to lack of GA/SA and complications related to GA/SA; 3) dosimetric analysis not according to recent GEC‐ESTRO guidelines,^(20)^ which will be increasingly followed in future. This was not possible in our study since half of the patients (AG patients) were treated before 2007 when the GEC‐ESTRO guidelines were not widely adopted in practice. Despite these limitations, the findings of our study have highlighted the unexplored impact of anesthesia on HDR‐ICBT dosimetry, an equally important concern with any brachytherapy procedure, and would certainly interest the readers.

## V. CONCLUSIONS

Although based on conventional 2D dosimetry planning, our study has demonstrated that avoiding GA/SA does not result in inferior ICBT dosimetry. Therefore, for routine ICBT applicator insertion in a high volume center, use of GA/SA may be safely avoided and reserved for patients who are uncooperative due to the fear of pain or discomfort. This approach would reduce the burden of anesthesia team, and time gained due to avoidance of GA/SA would result in treating larger number patients with cervical cancer.

## References

[1] Kavanagh BD , Perez CA . Uterine cervix. In: Halperin EC, Parez CA, Brady LW, editors. Perez and Brady's principles and practice of radiation oncology, 5th edition Philadelphia, PA: Lippincott Williams & Wilkins; 2008 p.1533–609.

[2] Viswanathan AN , Petereit DG . Gynecologic brachytherapy. In: Devlin PM, editor. Brachytherapy: applications and technique, 1st edition Philadelphia, PA: Lippincott Williams & Wilkins; 2007 p.223–67.

[3] Grigsby P and Perez C . Radiotherapy alone for medically inoperable carcinoma of the cervix: stage IA and carcinoma in situ. Int J Radiat Oncol Biol Phys 1991;21(2):375–78. 1905690 10.1016/0360-3016(91)90785-3

[4] Biswal BM , Lal P , Rath GK , Mohanti BK . Hemostatic radiotherapy in carcinoma of the uterine cervix. Int J Gynaecol Obstet. 1995;50(3):281–285. 8543112 10.1016/0020-7292(95)02454-k

[5] Nag S , Erickson B , Thomadsen B , Orton C , Demanes JD , Petereit D . The American brachytherapy society recommendations for high‐dose‐rate brachytherapy for carcinoma of the cervix. Int J Radiat Oncol Biol Phys. 2000;48(1):201–11. 10924990 10.1016/s0360-3016(00)00497-1

[6] Viswanathan AN , Creutzberg C , Craighead P , et al. International brachytherapy practice patterns: a survey of the Gynecologic Cancer Intergroup (GCIG). Int J Radiat Oncol Biol Phys. 2012;82(1):250–55. 21183288 10.1016/j.ijrobp.2010.10.030 PMC3489266

[7] Lim KH , Lu JJ , Wynne CJ , et al. A study of complications arising from different methods of anesthesia used in high‐dose‐rate brachytherapy for cervical cancer. Am J Clin Oncol. 2004;27(5):449–51. 15596908 10.1097/01.coc.0000128723.00352.ad

[8] ICRU . Dose and volume specification for reporting intracavitary therapy in gynecology. ICRU Report 38. Bethesda, MD: ICRU 38; 1985.

[9] Aronowitz JN , Aronowitz SV , Robison RF . Classics in brachytherapy: Margaret Cleaves introduces gynecologic brachytherapy. Brachytherapy. 2007;6(4):293–97. 17991626 10.1016/j.brachy.2007.08.009

[10] Chen HC , Leung SW , Wang CJ , et al. Local vaginal anesthesia during high‐dose‐rate intracavity brachytherapy for cervical cancer. Int J Radiat Oncol Biol Phys. 1998;42(3):541–44. 9806512 10.1016/s0360-3016(98)00243-0

[11] Benrath J , Kozek‐Langenecker S , Hupfl M , Lierz P , Gustorff B . Anaesthesia for brachytherapy – 51/2 yr of experience in 1622 procedures. Br J Anaesth. 2006;96(2):195–200. 16377650 10.1093/bja/aei301

[12] Smith MD , Todd JG , Symonds RP . Analgesia for pelvic brachytherapy. Br J Anaesth. 2002;88(2):270–76. 11878659 10.1093/bja/88.2.270

[13] Roessler B , Six LM , Gustorff B . Anaesthesia for brachytherapy. Curr Opin Anesthesiol. 2008;21(4):514–18. 10.1097/ACO.0b013e32830413cb 18660663

[14] Janaki MG , Nirmala S , Kadam AR , Ramesh BS , Sunitha KS . Epidural analgesia during brachytherapy for cervical cancer patients. J Cancer Res Ther. 2008;4(2):60–63. 18688120 10.4103/0973-1482.40825

[15] Anker CJ , O'Donnell K , Boucher KM , Gaffney DK . Effect ofbrachytherapy technique and patient characteristics on cervical cancer implant dosimetry. Med Dosim. 2013;38(4):430–35. [Epub ahead of print] 23973016 10.1016/j.meddos.2013.06.001

[16] Nag S , Chao C , Erickson B , et al. The American Brachytherapy Society recommendations for low‐dose‐rate brachytherapy for carcinoma of the cervix. Int J Radiat Oncol Biol Phys. 2002;52(1):33–48. 11777620 10.1016/s0360-3016(01)01755-2

[17] Viswanathan AN and Thomadsen B . American Brachytherapy Society consensus guidelines for locally advanced carcinoma of the cervix. Part I: general principles. Brachytherapy. 2012;11(1):33–46. 22265436 10.1016/j.brachy.2011.07.003

[18] Bhanabhai H , Samant R , E C , Grenier L , Lowry S . Pain assessment during conscious sedation for cervical cancer high‐dose‐rate brachytherapy. Curr Oncol. 2013;20(4):e307–e310. Available from: https://doi.org/10.3747/co.20.1404 23904769 10.3747/co.20.1404 PMC3728059

[19] Lanciano RM , Pajak TF , Martz K , Hanks GE . The influence of treatment time on outcome for squamous cell cancer of the uterine cervix treated with radiation: a patterns‐of‐care study. Int J Radiat Oncol Biol Phys. 1993;25(3):391–97. 8436516 10.1016/0360-3016(93)90058-4

[20] Haie‐Meder C , Pötter R , Van Limbergen E , et al. Recommendations from Gynaecological (Gyn) GEC‐ESTRO Working Group (I): concepts and terms in 3D image based 3D treatment planning in cervix cancer brachytherapy with emphasis on MRI assessment of GTV and CTV. Radiother Oncol. 2005;74(3):235–45. 15763303 10.1016/j.radonc.2004.12.015

